# Is early appendectomy in adults diagnosed with acute appendicitis mandatory? A prospective study

**DOI:** 10.1186/s13017-018-0221-2

**Published:** 2019-01-11

**Authors:** Salma Abu Foul, Ella Egozi, Ahmad Assalia, Yoram Kluger, Ahmad Mahajna

**Affiliations:** Department of General Surgery, Rambam Health Care Campus, The Technion - Institute of Technology, P.O. Box 9602, 31096 Haifa, Israel

**Keywords:** Acute appendicitis, Appendectomy, Postoperative complications

## Abstract

**Introduction:**

Prompt appendectomy has long been the standard of care for acute appendicitis in order to prevent complications such as perforation, abscess formation, and diffuse purulent or fecal peritonitis, all resulting in increased morbidity and even mortality. Our study was designed to examine whether the time from the beginning of symptoms to operation correlates with the pathological degree of appendicitis, incidence of postoperative complications, or increased length of hospital stay.

**Methods:**

A prospective study of 171 patients who underwent emergent appendectomy for acute appendicitis in the course of 2 years was conducted in a single tertiary medical center. The following parameters were monitored and correlated: demographics, time from the onset of symptoms until the arrival to the emergency department (patient interval (PI)), time from arrival to the emergency department (ED) until appendectomy (hospital interval (HI)), time from the onset of symptoms until appendectomy (total interval (TI)), physical examination, preoperative physical findings, laboratory data, pathologic findings, complications, and length of hospital stay.

**Results:**

The degree of pathology and complications were analyzed according to the time intervals. The time elapsed from the onset of symptoms to surgery was associated with higher pathology grade (*p* = 0.01). We found that longer time from the onset of symptoms to hospital arrival correlates with higher pathology grade (*p* = 0.04), while there was no correlation between the hospital interval and pathology grade (*p* = 0.68). A significant correlation was found between the pathology grade and the incidence of postoperative complications as well as with increased length of hospital stay (*p* = 0.000).

**Conclusion:**

Time elapsed from the symptom onset to appendectomy correlates with increased pathology grade and complication rate. This correlation was not related to the HI. Since the HI in our study was short, we recommend an early appendectomy in adults in order to shorten the TI and the resulting complications.

## Introduction

Acute appendicitis is one of the most common surgical emergencies encountered in the ED. This condition may be associated with complications and significant rise in morbidity and even mortality if diagnosis and treatment are delayed [1]. Since the first appendectomy in 1883, early appendectomy has been advocated for acute appendicitis [2–4]. The course of conservative therapy of acute appendicitis with antibiotics has been accepted in the pediatric population [5–7]. This concept of conservative therapy challenged the historical paradigm of emergent appendectomy in the adult population [8–10].

Despite the fact that conservative treatment with antibiotics has been shown to be safe also in adults, laparoscopic appendectomy remains the standard of care for acute appendicitis in adults [11–13]. This operation is frequently done by junior staff and can be performed in selected cases as an ambulatory surgery [14, 15].

The duration of the inflammation of the appendix is related to the risk of perforation [16–20]. Time periods between the onset of symptoms, medical assessment, and treatment are important. Previous studies revealed that in-hospital delay increases the risk of perforation in adults with appendicitis. Perforation was associated with a higher complication rate and increased length of hospital stay [21, 22]. On the other hand, in recent studies, delayed appendectomy, from 12 to 36 h from the symptom onset, was acceptable [3, 23–25].

The aim of this study is to determine the relationship between the time of the symptom onset to surgery (patient interval (PI) and hospital interval (HI)) in regard to pathological severity, complications, and hospital stay in patients with acute appendicitis.

## Methods

A prospective study including all patients with the diagnosis of acute appendicitis at one tertiary medical center in the course of 2 years was designed. The study was approved by the Helsinki Committee at Rambam Medical Center. Exclusion criteria included patients younger than 16 years, patients who could not recollect the exact timing of symptom onset, and patients who had no appendicitis in the pathology examination. The diagnosis of acute appendicitis was confirmed by an ultrasound or computed tomography scan images prior to surgery in all patients.

A personal interview in regard to the time from the onset of symptoms to the hospital arrival (PI) was undertaken and recorded.

At surgery, the surgeon described the macroscopic appearance and graded the appendicitis, according to the scale presented by Ditillo and colleagues in 2006 [4]: simple appendicitis was graded G1, phlegmonous graded G2, gangrenous graded G3, and perforated graded G4. All specimens were examined by a pathologist.

Postoperative complication rate and hospital stay were recorded. Parameters were monitored including the time from hospital arrival to surgery (HI) and time from the symptom onset to surgery (total interval (TI)).

Antibiotics were administered for all patients immediately after the diagnosis of appendicitis. Patients with an intraoperative finding of intra-abdominal abscess or perforated appendicitis were given extended courses of antibiotics postoperatively. Appendectomies at our hospital are always initiated with a laparoscopic approach. Open conversion is done at any time when the surgeon judges that the laparoscopic procedure is unsafe or no longer possible.

### Statistical analysis

Pearson’s chi-square test was applied for categorical parameters, and the Kruskal-Wallis test and Mann-Whitney test for continuous variables. A *p* value of < 0.05 was considered statistically significant. Data was generated with SPSS software (version 15.01.1, SPSS Inc., 2006). Descriptive statistics and graphical methods were used to characterize the date using Microsoft Office Excel.

## Results

### Demographics

One hundred seventy-one patients were included in our study: 112 men (65.5%) and 59 women (34.5%). Age ranged from 16 to 86 years, with a mean age of 33 years. There was no correlation between gender and the degree of pathology (*p* = 0.46), while advanced pathology grade was associated with advanced age (*p* = 0.001) (Table 1).

**Table 1:** Relationship between demographic parameters and degree of pathology

	G1	G2	G3	G4	Total
Age (average - years)	26.29	33.71	39.12	47.43	32.98
Male (N)	27	63	17	5	112
Female (N)	21	28	8	2	59
Total	48	91	25	7	171

### Relationship between physical examination parameters and the degree of pathology

The mean temperature was 37.26 °C, ranging from 37.06 to 38.1 °C, with significant correlation to advanced pathological degree (*p* = 0.001). The highest mean temperature was 38.1 °C noted at grade 3.

The levels of WBC ranged from 12.7 to 14 thousand with a mean of 13.6. The correlation between leukocytosis and high pathological grade was significant (*p* = 0.028) as well. Advanced level was noted at grade 3 (Table 2).

**Table 2:** Relationship between physical examination parameters and the degree of pathology

	Degree of pathology				
	G1	G2	G3	G4	*p* value
Number of patients	48	90	25	7	
Fever (°C)	37.06	37.11	38.1	37.26	< 0.001
WBC	12,700	13,500	14,060	13,590	= 0.028
Abdominal tenderness (%)	96	98	96	100	= 0.863
Peritonitis (%)	65	68	72	100	= 0.293

*NR* number of patients, *°C* Celsius

Our data shows that the parameters of physical examination including abdominal tenderness and peritonitis were not correlated to the advanced degree of pathology. The percentage of abdominal tenderness among our patients ranged from 96 to 100%, with a calculated *p* value of 0.863. While peritonitis was noted in 65 to 100%, the calculated *p* value was 0.29 (Table 2).

### Relationship between the time of surgery to the degree of pathology

The prevalence of advanced pathology positively correlated with prolonged PI, ranging from 30.17 to 49.11 h. For grade 1, the average time was 30.17 h, while the average time for grade 4 was 49.11 h, with a *p* value of 0.04.

The same positive relation was found for the TI, time range from 42.29 to 63 h, while for grade 1, the interval noted was 42.29 h, and for grade 4 was 63 h, with a *p* value of 0.01.

At our study, the HI did not correlate with the degree of pathology; the average time for all grades was 12.37 h, range from 12.13 to 13.86 h, with a *p* value of 0.68 (Table 3).

**Table 3:** Relationship between the timing of surgery and degree of pathology

	G1	G2	G3	G4	*p* value
PI (h)	30.17	31.25	47.76	49.11	= 0.04
HI (h)	12.13	11.8	11.72	13.86	= 0.681
TI (h)	42.29	43.05	59.48	63	= 0.01

*PI* patient interval, *HI* hospital interval, *TI* total interval, *h* hours

### Relationship between postoperative hospital stay, complication, and the degree of pathology

Hospital stay and the rate of complication increased according to higher pathological degree with a *p* value of 0.000 for the two parameters (Table 4). For grade 1, the complication rate was 2%, increased according to the advanced grade, reaching 100% for grade 4. For hospital stay, the average days in the hospital for grade 1 were 3.21 days, while 9 days for grade 4.

**Table 4:** Relationship between postoperative hospital stay and complications

	G1	G2	G3	G4	*p* value
Number of patients	48	90	25	7	
Hospital stay (days)	3.21	3.6	5.08	9	= 0.00
Complications (%)	2	8	48	100	= 0.00

## Discussion

The timing of the onset of the symptoms of appendicitis is important in deciding when to perform acute care surgery. Delayed surgery from the symptom onset is associated with worse outcomes for the vast majority of the surgical emergencies [25–27]. As noted in retrospective studies conducted in the adult population, acute appendicitis is a time-dependent disease in regard to severity and complication rate [6]. Delay in the overall timing of treatment increased the risk of progression of pathology and postoperative complications [4]. Such findings are contradicted with studies on child population, suggesting antibiotic treatment as a mean of therapy [5–7]. In adults, appendectomy remains the most effective and safe treatment for acute appendicitis when compared with antibiotic treatment [2, 28–30]. The urgency of the operation is still controversial.

In accordance to known data, at this study, the main population was young men, with the mean age of 33 years old. A positive correlation was found between the advanced age and advanced degree of pathology. This relation might be contributed to the longer time to diagnosis in the older population known from the different octogenarian studies; this was not monitored at the current study and needs further research.

Pathological degree correlates strongly with prolonged time from the symptom onset to the hospital arrival and from the symptom onset to appendectomy; such findings correlate with previse conclusions of retrospective studies [28, 29]. Suggesting the need for expeditious appendectomy once the diagnosis was confirmed.

The hospital interval, from diagnosis to surgery, was not found to be a statistically significant parameter at the current study, in contrast with previous studies [4, 21]. Such difference may be contributed to relatively short time from diagnosis to surgery at our center (11.8–13.8 h) which is at the lower time limit suggested in the literature, 12–36 h [3, 23]. It might be also contributed to the prospective nature of our study. Though the in-hospital time was not found to be a factor of pathological severity at this study, the debate about the acceptable time is yet to be determined.

Although PI showed statistical significance, it is not easy to shorten this time period directly. Therefore, it is recommended that clinicians focus on shortening the total interval. Reducing PI might be accomplished through education and dissemination of publicity.

According to our study, higher pathological degree was in correlation to a longer hospital stay and to a higher rate of complication. Considering our data which demonstrates positive correlation between advanced pathology grade and the total time interval and the patient interval, an urgent appendectomy is recommended, though such surgery might be delayed according to the hospital capability while the patient is hospitalized with antibiotic therapy awaiting surgery.

Surprisingly, at our study, abdominal tenderness and signs of peritonitis were not correlated with the degree of pathological severity, in contrast to previous studies [4]; the deference might be the prospective nature of this study.

## Conclusion

Based on the positive relation between the onset of symptoms to appendectomy, pathological severity, and complication rate, we advise early appendectomy according to the hospital capability; these findings are consistent with the conclusions of the World Society of Emergency Surgery study group initiative [31]. A large-sample prospective study should be conducted to set the optimal time for surgery since the diagnosis of appendicitis.

## Abbreviations

ED: Emergency department
HI: Hospital interval
PI: Patient interval
TI: Total interval
